# Ginsenoside Compound K Induces Adult Hippocampal Proliferation and Survival of Newly Generated Cells in Young and Elderly Mice

**DOI:** 10.3390/biom10030484

**Published:** 2020-03-23

**Authors:** Jung-Mi Oh, Jae Hoon Jeong, Sun Young Park, Sungkun Chun

**Affiliations:** 1 Department of Physiology, Chonbuk National University Medical School, Jeonju-si, Jeollabuk-do 54907, Korea; biojmi@jbnu.ac.kr (J.-M.O.); jjh8690624@gmail.com (J.H.J.); qirsjm@naver.com (S.Y.P.); 2 Brain Korea 21 Plus Program, Chonbuk National University Medical School, Jeonju-si 54907, Korea

**Keywords:** ginsenoside CK, neurogenesis, cell proliferation, neuroprotection

## Abstract

Cognitive impairment can be associated with reduced adult hippocampal neurogenesis, and it may contribute to age-associated neurodegenerative diseases such as Alzheimer’s (AD). Compound K (CK) is produced from the protopanaxadiol (PPD)-type ginsenosides Rb1, Rb2, and Rc by intestinal microbial conversion. Although CK has been reported as an inducing effector for neuroprotection and improved cognition in hippocampus, its effect on adult neurogenesis has not been explored yet. Here, we investigated the effect of CK on hippocampal neurogenesis in both young (2 months) and elderly (24 months) mice. CK treatment increased the number of cells co-labeled with 5-ethynyl-2′-deoxyuridine (EdU) and proliferating cell nuclear antigen (PCNA); also, Ki67, specific markers for progenitor cells, was more expressed, thus enhancing the generation of new cells and progenitor cells in the dentate gyrus of both young and elderly mice. Moreover, CK treatment increased the number of cells co-labeled with EdU and NeuN, a specific marker for mature neuron in the dentate gyrus, suggesting that newly generated cells survived and differentiated into mature neurons at both ages. These findings demonstrate that CK increases adult hippocampal neurogenesis, which may be beneficial against neurodegenerative disorders such as AD.

## 1. Introduction

Since adult hippocampal neurogenesis in the dentate gyrus [1] has first been reported, it has been considered a main contributor to age-related cognitive impairments [2] and to neurodegenerative diseases such as Alzheimer’s disease (AD) [3]. Aging leads to several symptoms, such as inflammation-induced brain metabolic changes, neurovascular defects, memory loss, and increased anxiety level in mice [4]. Furthermore, aging both reduces adult hippocampal neurogenesis [5,6] and increases the risk factors for AD [7,8,9]. Indeed, age-dependent neurogenesis reduction [5,10,11] leads to decreased learning and memory function [12]. Therefore, any therapy aiming to increase adult hippocampal neurogenesis could be an important strategy to mitigate age-related neurodegenerative diseases such as AD.

Ginsenoside compound K (CK) is conversion from protopanaxadiol (PPD)-type ginsenosides Rb1, Rb2, and Rc by intestinal microbiota [13]. Previous researches reported that CK exhibits beneficial pharmacological effects, including antidiabetic [14], anti-inflammatory [15], anticancer [16], and antiaging [17] activity. Recent evidences suggest that CK has a role in neuroprotection and in cognitive improvement. For instance, CK protects neurons in the stroke mouse model by reducing microglial activation through its anti-inflammatory activity [18]. Also, chemotherapy-induced cognitive impairment was mitigated by CK administration in mice [19]. In vascular dementia rats, CK cleared the beta-amyloid deposition, resulting in the attenuation of both neuronal damage and cognitive deficits [20]. In addition, 10 mg/Kg CK was successfully used in mice to reduce scopolamine-induced memory impairment and negative effects of reactive oxygen species via the induction of Nrf2-mediated antioxidant enzyme [21]. Although previous studies regarding the neuroprotective beneficial effects of CK have been reported, the direct evidence regarding the effect of CK on adult hippocampal neurogenesis is still unknown. Hence, in this study, we investigated the potential of CK to modulate adult hippocampal neurogenesis.

## 2. Materials and Methods

### 2.1. Animals

All mouse care and experimental procedures were approved by the Institutional Animal Care and Use Committee of the Chonbuk National University (permit number: CBNU-2018-042, Approval date: 05 June 2018). Two-month-old (-mo) and 24-mo male mice were purchased from Jackson Laboratories (Bar Harbor, ME, USA). Animals were kept grouped in cages at 22 °C with a 12:12-h light–dark cycle and fed a standard chow diet with ad libitum access to water.

### 2.2. Compound K Administration

Ginsenosides compound K was obtained from the laboratory of Ace EMzyme, Hankyung National University, South Korea. Compound K (CK) was dissolved in 100% dimethyl sulfoxide (DMSO) and then diluted daily in 2-hydroxypropyl-cyclodextrin (cat. no. H5784; Invitrogen). For 3 consecutive days, 2-mo and 24-mo mice were injected intraperitoneally either diluted CK (5, 10, or 15 mg/Kg) or vehicle (0 mg/Kg).

### 2.3. EdU Administration

To determine the cell proliferation rate induced by CK, on the last CK administration day, all mice received an intraperitoneal injection of phosphate-buffered saline (PBS)-EdU (100 mg/Kg, ab146186, Abcam, Cambridge, Massachusetts, UK). Mice were then sacrificed after 24 h. For neuronal survival analysis, EdU was injected for 3 consecutive days after the last CK injection, and all remaining mice were sacrificed after 4 weeks.

### 2.4. Perfusion

At the end of the experiment, all mice were perfused with 0.1 M PBS containing heparin (1000 u/mL; cat. no. H3393; Sigma-Aldrich; Merck KGaA, Darmstadt, Germany) and prefixed with 4% paraformaldehyde (PFA) in 0.1 M PBS. All brains were postfixed in a 4% PFA solution for 24 h at 4 °C, dehydrated in a 30% sucrose solution at 4 °C for 72 h, and immediately frozen at −80 °C. Coronal sections (40 μm) were cut with a cryostat (Leica CM 1860, Leica Biosystems Inc., Chicago, IL, USA), and slices were stored in cryoprotectant (25% ethylene glycol and 25% glycerin) in 0.05 M phosphate buffer at −20 °C.

### 2.5. Immunohistochemistry

Every sixth serial section was tested for cell proliferation with the Click-iT EdU Cell Proliferation Kit for Imaging, Alexa Fluor 488 dye (cat. no. C10337; Invitrogen, Gaisburg, MD, USA) according to the manufacturer’s instructions. Briefly, the free-floating sections were washed 3 times in 0.1 M PBS containing 3% bovine serum albumin (BSA), permeabilized with 0.5% Triton-X 100 in PBS for 20 min, and rinsed with 3% BSA in PBS. Then, the sections were incubated with the Click-iT reaction buffer for 30 min at room temperature (RT), washed 3 times in PBS, mounted on a slide-glass using Vectashield antifade mounting medium (cat. no. H-1000; Vector Laboratories, Burlingame, CA, USA), and covered with a coverslip. For EdU and immunohistochemistry double labeling, the sections were incubated in 1 × Target Retrieval Solution (Dako, cat# S1699) at 95 °C for 5 min prior to the EdU staining procedure. After EdU staining, sections were incubated in blocking solution (5% BSA and 0.1% Triton X-100 in PBS) for 2 h at RT and then incubated with anti-proliferating cell nuclear antigen (anti-PCNA) (1:1000; cat. no. 2586; Cell Signaling Technology, Danvers, MA, USA), anti-Ki67 (1:1000; cat. no. ab15580; Abcam), or anti-NeuN (1:1000; cat. no. 24307; Cell Signaling Technology) antibodies for 24 h at 4 °C. Sections were then washed 3 times in PBS and incubated with Alexa 594 anti-mouse IgG (1:1000; cat. no. ab150116; Abcam) or Alexa 594 anti-rabbit IgG (1:1000; cat. no. ab150080; Abcam) for 2 h at RT. Tissues were washed, dried, and mounted with mounting medium. Images were acquired using a Zeiss LSM 880 super resolution confocal microscope.

### 2.6. Immunoblotting

Radioimmunoprecipitation assay (RIPA) buffer (cat. no. R2002; Biosesang, Seongnam-si, South Korea) containing proteinase inhibitors (cat. no. G652A; Promega, South Korea) was used to obtain total lysates from the hippocampus of 2-mo and 24-mo C57BL/6 mice injected with vehicle or CK. Total lysates (20 μg) were separated on 12% SDS-PAGE gels and transferred to polyvinylidene fluoride (PVDF) membranes and then kept in 5% skim milk blocking buffer for 2 h at RT. Anti-brain-derived neurotrophic factor (anti-BDNF) (1:1000; cat. no. ab108319; Abcam), anti-pAkt (Ser473) (1:1000; cat. no. 4060; Cell Signaling Technology), anti-Akt (1:1000; cat. no. 9272; Cell Signaling Technology), anti-pERK1/2 (1:1000; cat. no. 9101; Cell Signaling Technology), anti-ERK1/2 (1:1000; cat. no. 4696; Cell Signaling Technology), and anti-GAPDH (1:5000; cat. no. AP0066; Bioworld Technology) antibodies diluted in TBST (Tris buffered saline Tween 20) buffer containing 5% BSA, which was used for the membranes’ incubation for 24 h at 4 °C. The membranes were then washed in TBST buffer and probed with anti-rabbit IgG, horseradish peroxidase (HRP)-linked or anti-mouse IgG, HRP-linked secondary antibodies (1:3000; cat. no. 7074 and 7076; Cell Signaling Technology) for 2 h at RT. All membranes were washed in TBST buffer and developed using Clarity Western ECL Substrate (cat. no. 1705060; BIO RAD, Hercules, CA, USA).

### 2.7. Quantitative Reverse Transcription PCR

For qRT-PCR analysis of neurotrophin 3 (NT3) and brain-derived neurotrophic factor (BDNF) genes, total RNA was isolated from hippocampus using the Trizol reagent (Thermo Fisher Scientific, Inc., 15596026) and then first-strand cDNA was synthesized using GoScript Reverse Transcription System (cat. no. A5001; Promega). Real-time qPCR was performed with 7900 HT fast Real-Time PCR System (Applied Biosystems) using SYBR Green I Master Mix (cat. no. RT501M; Enzynomics). Each qPCR reaction was prepared in a final volume of 10 μL, containing 2 μL cDNA and 5 μL of SYBR Green Master mix with a different primer pair (1 μL, 10 pmole/μL). Beta-actin was used as an internal control for the quantification of each sample. The primer pairs employed are F5′-CAAAGGGATCGTTGGAGGTGA-3′ and R5′- GTTCGGTCAT TCAGTCTCGC-3′ for NT3, F5′-TCATACTTCGGTTGCATGAAGG-3′ and R5′-AGACCTCTC GAACCTGCCC-3′ for BDNF, and F5′-CCTGACAGACTACCTCATGAAG-3′ and R5′- CCATC TCTTGCTCGAAGTCTAG-3′ for beta-actin. Relative gene expression was determined using the ^ΔΔ^Ct method. Relative mRNA expression levels were presented as the fold change compared with the 2-mo mice vehicle group.

### 2.8. Statistical Analysis

All statistics were performed with GraphPad Prism (version 5.01.) for windows (Graphpad Software, San Diego, CA, USA). Data were expressed as mean ± standard error of the mean (SEM). Statistical significance was assessed using an unpaired t-test. Results with *p* < 0.05 were considered significant.

## 3. Results

### 3.1. CK Increases the Number of Newly Born Cells in the Dentate Gyrus

**Figure 1:**
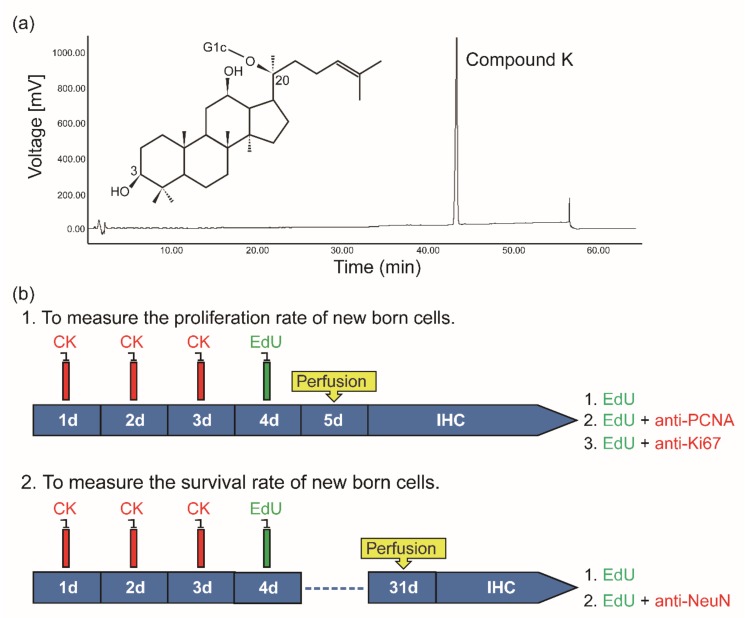
(**a**) Chemical structure of Compound K (CK) and high-performance liquid chromatography (HPLC) analysis of the transformation of CK: The chromatographic peak was identified by comparison with the reference compounds. (**b**) Experimental design. In experiment 1: mice were injected with 5-ethynlyl-2′-deoxyuridine (EdU, 100 mg/Kg) once after 3 consecutive CK intraperitoneal injections and sacrificed on day 5 or on day 31 for proliferation or survival studies, respectively. Antibodies for EdU, proliferating cell nuclear antigen (PCNA), and Ki67 were used to measure the proliferation of newly born cells, while antibodies for EdU and NeuN were used to measure the survival rate of newly born cells.

We first purified compound K (>97%, molecular structure in Figure 1A) by acid-heat treatment of PPD-type ginsenoside extracts from Korean ginseng (Panax ginseng). To determine the effect of CK on cell proliferation in young and elderly mice, both mice groups were injected with various concentration of CK (0 mg/Kg, 5 mg/Kg, 10 mg/Kg, and 15 mg/Kg) for 3 consecutive days and then with EdU (Figure 1b). Twenty-four h after EdU injection, all mice were transcardially perfused and the brain was sectioned for histological study using a cryostat (40 μm).

**Figure 2:**
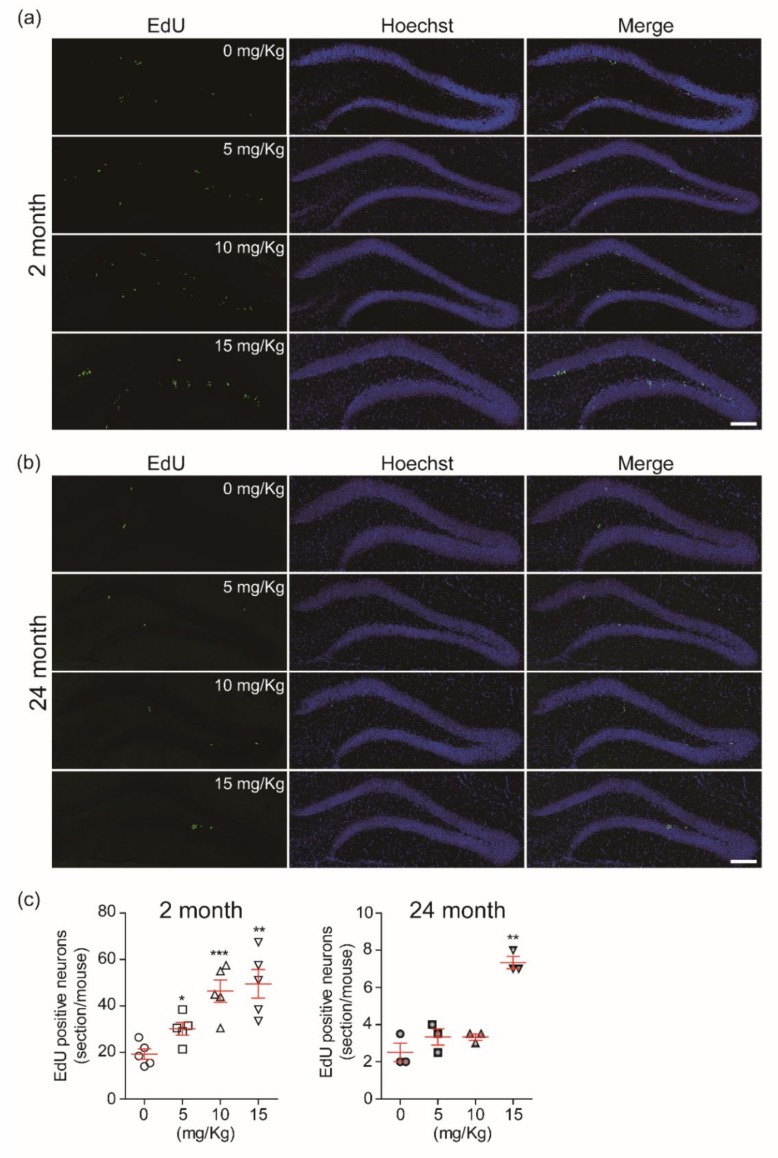
Compound K (CK) induces the production of new cells in the dentate gyrus (DG) of hippocampus: Images of confocal microscopy showing 5-ethynyl-2′-deoxyuridine (EdU)-labeled cells in the dentate gyrus of 2-month-old (-mo) (**a**) and 24-mo mice (**b**). All C57BL/6 mice were intraperitoneally injected with EdU (5 mg/Kg, 10 mg/Kg, and 15 mg/Kg) or vehicle for 3 consecutive days. (**c**) Summary plots showing that CK promotes the generation new cells in a dose-dependent manner (*n* = 5 in 2-mo; *n* = 3 in 24-mo mice, respectively). All data are represented as the mean ± standard error of the mean (SEM). *p < 0.05, **p < 0.01, and ***p < 0.001 vs. vehicle treated groups (unpaired *t*-test).

The total number of EdU-incorporated cells was counted in the subgranular zone of the dentate gyrus. The CK dose-dependently increased the number of EdU-incorporated newborn cells in the dentate gyrus of young mice (Figure 2a,c). However, the production of new cells in 24-mo mice was significantly increased only with 15 mg/Kg CK (Figure 2b,c). These results show that the number of new cells does decrease by aging, but this trend could be slowed by treatments with more concentrated ginsenoside CK.

### 3.2. CK Enhances the Proliferation of Newly Born Cells in the Dentate Gyrus

**Figure 3:**
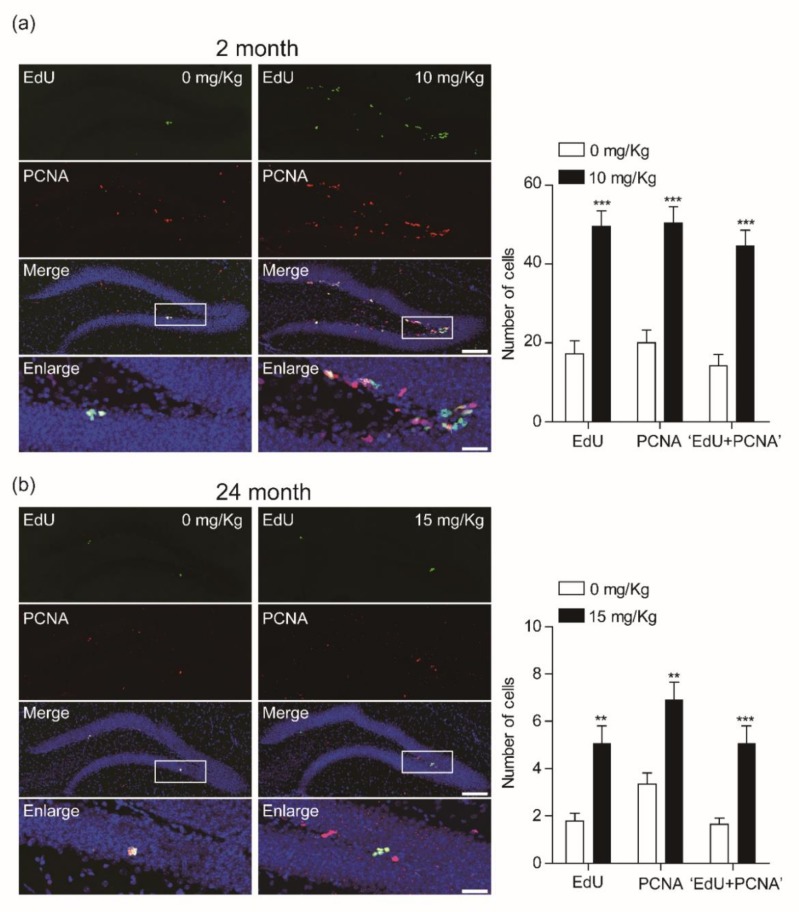
Compound K (CK) increases 5-ethynyl-2′-deoxyuridine (EdU)+/PCNA+ cells as well as PCNA+ cells in the DG of hippocampus. (**a**) Co-expression of PCNA in the EdU-labeled newborn cells in the dentate gyrus (DG) of 2-mo mice (0 mg/Kg, *n* = 5 mice; 10 mg/Kg, *n* = 4 mice; ***p < 0.001, unpaired *t*-test). Scale bar = 100 μm (merge) or 20 μm (enlarge). (**b**) Image of confocal microscopy showing expression of PCNA in the EdU-labeled newborn cells of DG of 24-mo mice. Plots showing that CK increases PCNA positive cells in EdU-labeled newborn cells (0 mg/Kg, *n* = 7; 15 mg/Kg, *n* = 7; **p < 0.01, ***p < 0.001, unpaired *t*-test). Scale bar = 100 μm (merge) or 20 μm (enlarge).

Given that CK showed potent effects on newly born cell production in the dentate gyrus of both age groups, we next investigated its effects using double immunofluorescence staining with the anti-proliferating cell nuclear antigen (PCNA), which is a marker for cell proliferation. We found that 10 mg/Kg CK significantly increased both PCNA-labeled cells and EdU-PCNA double-labeled cells in the dentate gyrus of hippocampus (Figure 3).

**Figure 4:**
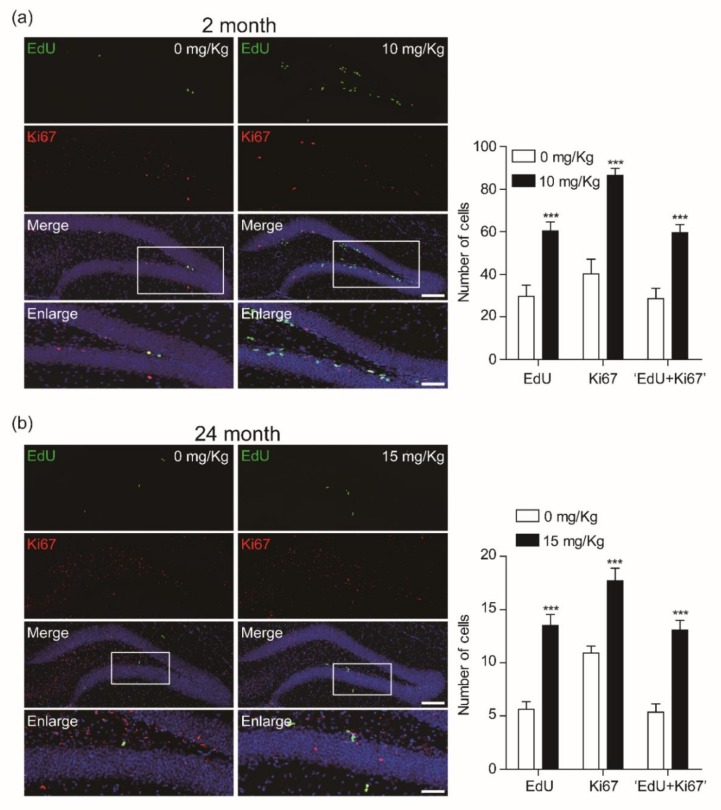
Compound K (CK) induces the number of co-labeled cells with 5-ethynyl-2′-deoxyuridine (EdU) incorporation and anti-Ki67 in the dentate gyrus (DG) of hippocampus. Confocal images showing EdU+/Ki67+ cells in the DG of (**a**) 2-mo (0 mg/Kg, *n* = 5; 10 mg/Kg, *n* = 6) and (**b**) 24-mo mice (0 mg/Kg, *n* = 7; 15 mg/kg, *n* = 7). The summary plots show quantification of positive cells for EdU, Ki67, or EdU/Ki67 in 2-mo or 24-mo mice with CK treatment. All data are represented as the mean ± standard error of the mean (SEM). ***p < 0.001 vs. vehicle treated groups (unpaired *t*-test). Scale bar = 100 μm (merge) or 20 μm (enlarge).

Ki-67 is also a classic marker for cellular proliferation and is preferentially expressed during the late G1, S, G2, and M phases of the cell cycle, whereas resting, non-cycling cells (G0 phase) lack Ki-67 expression. Also, it is reported that the Ki-67 antigen is a more specific marker for cell proliferation than PCNA [22]. We further confirmed the effect of CK on the proliferation of newborn cells in the dentate gyrus of hippocampus. Ten mg/Kg CK significantly enhanced the Ki-67 positive cells in the dentate gyrus of hippocampus as well as Ki-67 and EdU double-positive cells (Figure 4). These results suggest that CK enhances the proliferation rate of newborn cells, resulting in an increase of both PCNA and Ki-67-labeled EdU-positive cells.

### 3.3. CK Improves Adult Hippocampal Neurogenesis by Increasing Neuronal Survival

To investigate whether CK induces adult hippocampal neurogenesis through an increasing survival rate of newborn cells in the DG of hippocampus of both 2-mo and 24-mo mice, we performed immunohistochemistry for EdU and mature neuronal marker protein (NeuN) co-labeled neurons.

**Figure 5:**
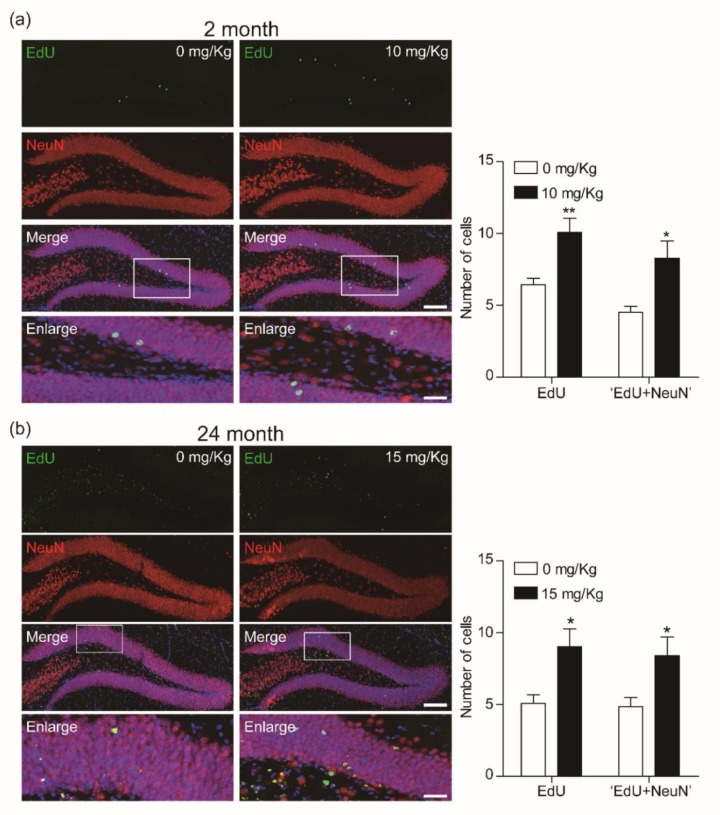
Compound K (CK) enhances the survival of newly generated cells in the dentate gyrus (DG) of hippocampus. Confocal images show that the survival rate of 5-ethynyl-2′-deoxyuridine (EdU)-labeled new born cells were enhanced by CK in the DG of (**a**) 2-mo (*n* = 6 in 0 mg/Kg and 10 mg/Kg, respectively) and (**b**) 24-mo mice (*n* = 6 in 0 mg/Kg; *n* = 5 in 15 mg/Kg). The plots showed a significant induction in co-expression of EdU and NeuN (*p < 0.05, **p < 0.01, unpaired *t*-test). Scale bar = 100 μm (merge) or 20 μm (enlarge).

Both age groups showed a significant increase of EdU cells when treated with CK (Figure 5a,b). Furthermore, CK increased the proportion of EdU-labeled cells that also expressed NeuN in both aged mice (Figure 5a,b), indicating that CK induces not only the survival of newborn cells but also differentiation into neurons. Thus, CK seems to facilitate adult hippocampal neurogenesis.

### 3.4. Adult Neurogenesis by CK is Mediated via BDNF Signaling

**Figure 6:**
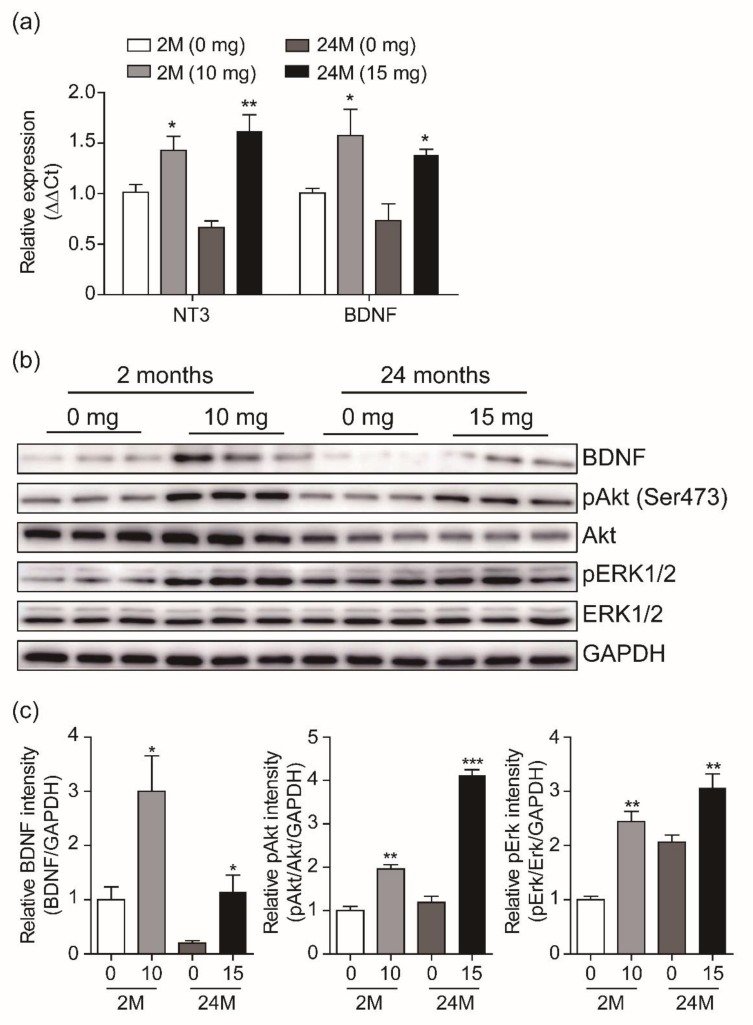
Beneficial effects of Compound K (CK) on adult neurogenesis is mediated via brain-derived neurotrophic factor (BDNF) signaling. (**a**) Summary plots showing neurotrophin-3 (NT3) and brain-derived neurotrophic factor (BDNF) mRNA expression in the dentate gyrus (DG) of hippocampus of 2-mo and 24-mo mice after CK (10 mg/Kg or 15 mg/Kg) or vehicle (0 mg/Kg) injection. Those expressions significantly increased after CK administration (*n* = 3, respectively, *p < 0.05, **p < 0.01, unpaired *t*-test). (**b**) Immunoblotting of total lysates from the DG after CK or vehicle treatment of both age groups. BDNF, Akt, and ERK signaling pathways for adult neurogenesis were screened (*n* = 3). (**c**) Summary plots showing the immunoblotting quantification for BDNF expression and its downstream targets: CK was sufficient to increase BDNF expression and the phosphorylation of Akt and ERK1/2, which are downstream targets of BDNF (*p < 0.05, **p < 0.01, ***p < 0.001, unpaired *t*-test).

**Figure 7:**
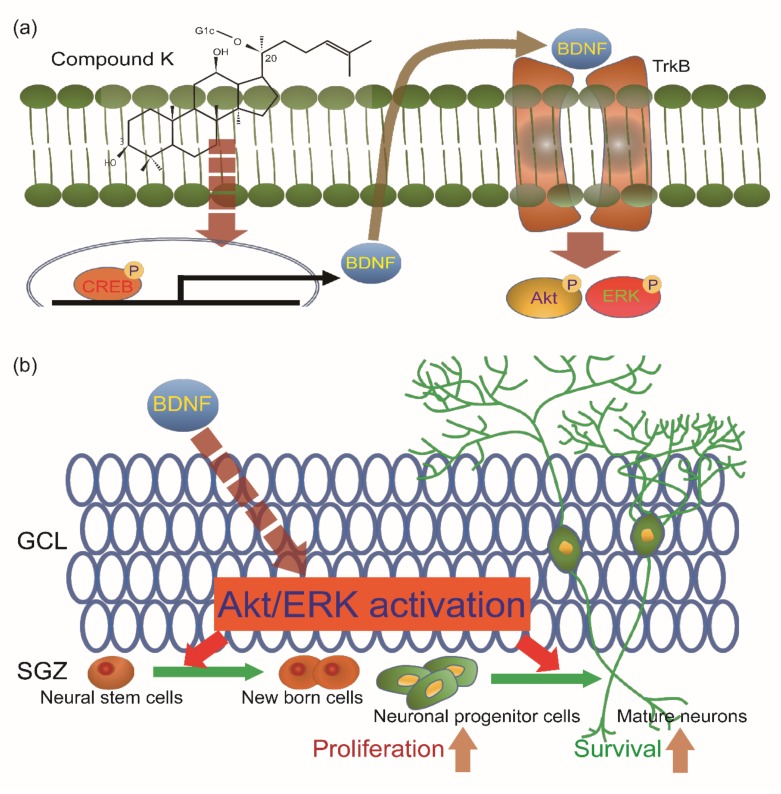
Schematic illustration of the proposed model: Compound K (CK) increases adult neurogenesis in the dentate gyrus (DG) of hippocampus by inducing BDNF signaling cascades. (**a**) CK increases the expression of BDNF and increased BDNF activates Akt and ERK signaling. (**b**) Activated Akt and ERK signals induced the proliferation and differentiation of newly born cells into progenitor cells and mature neurons. GCL, granule cell layer; SGZ, subgranular zone.

Neurotrophic factors and growth factors are well known as potent activators of adult hippocampal neurogenesis [23]. We thus examined whether CK-induced neurogenesis is mediated by those factors. Real-time qPCR was performed to determine the expression levels of neurotrophin-3 (NT3) and brain-derived neurotrophic factor (BDNF) in the dentate gyrus of the two age groups treated with CK or with the vehicle. After 3 days of treatment, the NT3 and BDNF mRNA levels in the CK-injected mice were significantly higher than those in the vehicle-injected mice (Figure 6a). In addition, the expression level of NT3 and BDNF was reduced in vehicle-injected 24-mo mice compared with vehicle-injected 2-mo mice, but both gene expression levels after CK administration were higher than those in the vehicle-injected 2-mo mice (Figure 6a). Next, we investigated the effect of CK administration on the influence of the intracellular BDNF signaling pathway over adult hippocampal neurogenesis. The activation of BDNF and its downstream signaling pathway leads neurogenesis through control of neuronal proliferation and survival [24,25]. As observed from our qRT-PCR data, BDNF expression was significantly increased by CK administration in 2-mo mice, while in CK-injected 24-mo mice, it was comparable to that of vehicle-injected 2-mo mice (Figure 6B,C). Furthermore, 10 mg/Kg and 15 mg/Kg CK were sufficient to induce the phosphorylation of Akt and ERK1/2, which are downstream targets of the BDNF signaling pathway in 2-mo and 24-mo mice, respectively (Figure 6b,c). These results indicate that CK-induced adult hippocampal neurogenesis is mediated by the BDNF signaling pathway (Figure 7).

## 4. Discussion

In this study, we provide the first evidence that ginsenoside compound K has the ability to induce the production, proliferation, and survival of newborn cells through the activation of BDNF signaling. Notably, CK administration was sufficient to increase hippocampal neurogenesis even in 24-mo mice, suggesting that CK can contrast age-related cognitive impairment by improving adult neurogenesis.

Compound K is known to have a neuronal protective role following chemotherapy treatment [19]. However, there was no direct evidence for the effect of CK on adult hippocampal neurogenesis so far. The decline of adult hippocampal neurogenesis by aging contributes to cognitive impairment and is associated with age-related neurodegenerative diseases such as AD [26,27,28]. Furthermore, exercise and dietary energy restriction enhance the adult hippocampal neurogenesis, improve learning and memory, and protect against age-related cognitive decline and AD [26,29,30]. Therefore, the enhancement of neurogenesis by exogenous factors could lead to novel therapeutic strategies for preventing cognitive impairment and AD. We found that CK administration increased newborn cells in the dentate gyrus of 24-mo mice, resulting in more newly generated EdU cells. Also, injections of 10 mg/Kg and 15 mg/Kg CK were sufficient to induce proliferation of newborn cells in 2-mo and 24-mo mice, respectively. In addition, CK significantly enhanced the differentiation of newly generated cells in the dentate gyrus of hippocampus into adult neurons. The rate of neuronal survival in CK-treated 24-mo mice was similar to its rate in 2-mo mice. These results suggest that CK has the ability to facilitate adult hippocampal neurogenesis under declining neurological conditions such as age-related cognitive impairment.

Neurotrophic factors such as BDNF and growth factors including neurotrophin-3 (NT3) are important activators of adult neurogenesis [31,32,33,34]. Hence, we examined whether CK-mediated induction of neurogenesis in the dentate gyrus is associated with BDNF and NT3 mRNA levels. Indeed, CK administration significantly increased the mRNA expression of BDNF and NT3 in the dentate gyrus of 2-mo and 24-mo mice. Interestingly, the mRNA levels of BDNF and NT3 in the CK-injected 24-mo mice were higher than 2-mo young mice. Previous studies show that the upregulation of BDNF and NT3 improves cognitive function in AD [35,36], suggesting that CK administration may be able to prevent cognitive impairment. Given that CK administration was sufficient to increase the BDNF mRNA level, we next investigated whether the overexpression of BDNF induced by CK administration could affect the phosphorylation of Akt and ERK1/2, which are downstream targets of the BDNF signaling cascade. In 2-mo and 24-mo mice, CK administration increased BDNF expression, which is consistent with the qRT-PCR results and the phosphorylation of Akt and ERK1/2. In particular, the BDNF protein level in 24-mo mice was lower than those in 2-mo mice (vehicle group) but CK administration was able to restore the BDNF protein up to the young mice level. The BDNF/Trkb signaling pathway has an essential role in the regulation of adult hippocampal neurogenesis [37]. Furthermore, the PI3K-Akt signaling pathway and activation of ERK1/2 signaling play a critical role in neuronal differentiation and in the survival of newly generated cells in the dentate gyrus [38,39,40], suggesting that CK-mediated activation of BDNF signaling contributes to increase the adult hippocampal neurogenesis in elderly mice (Figure 7).

## 5. Conclusions

In conclusion, the administration of CK resulted in an enhancement of adult hippocampal neurogenesis in both elderly and young mice. Therefore, ginsenoside compound K can become a therapeutic agent able to prevent age-related cognitive impairments due to reduced adult neurogenesis. Further studies are required to analyze the molecular mechanism by which compound K is able to induce BDNF transcription; promoter assay may help discovering the transcription factors involved in this CK-mediated induction of neurogenesis.
